# HOXB7 accelerates the malignant progression of hepatocellular carcinoma by promoting stemness and epithelial-mesenchymal transition

**DOI:** 10.1186/s13046-017-0559-4

**Published:** 2017-06-24

**Authors:** Hong-bo Huan, Da-peng Yang, Xu-dong Wen, Xue-jiao Chen, Liang Zhang, Li-li Wu, Ping Bie, Feng Xia

**Affiliations:** 1 Institute of Hepatobiliary Surgery, Southwest Cancer Center, Southwest Hospital, Third Military Medical University, Chongqing, 400038 China; 2 Laboratory of Biotherapy of Cancer, Southwest Cancer Center, Southwest Hospital, Third Military Medical University, Chongqing, 400038 China

**Keywords:** Hepatocellular carcinoma, Homeobox B7, Stemness, Epithelial-mesenchymal transition, c-Myc, Slug

## Abstract

**Background:**

Homeobox B7 (HOXB7) has been identified associated with poor prognosis of hepatocellular carcinoma (HCC). However, the specific mechanism by which HOXB7 promotes the malignant progression of HCC remains to be determined.

**Methods:**

Immunohistochemistry (IHC) was used to detect the expression level of HOXB7 in 77-paired HCC tissue samples, and the correlation between HOXB7 and HCC prognosis was assessed. The location of HOXB7 was confirmed by immunofluorescence. Cell Titer-Blue assay was used to assess the proliferation of hepatoma cells. The stem-like properties of hepatoma cells were analysed by sphere formation and clone formation assays. The effect of HOXB7 on expression of cancer stem cell markers was evaluated. Transwell and wound-healing assays were performed to estimate the invasion and migration abilities of hepatoma cells. A xenograft tumor model was established in nude mice to assess the role of HOXB7 in tumor growth. Bioluminescence imaging was used to survey the effect of HOXB7 on the metastatic ability of hepatoma cells in vivo.

**Results:**

Higher expression of HOXB7 was detected in HCC tissues compared with noncancerous tissues and significantly associated with poor prognosis of HCC. In addition, HOXB7 knockdown suppressed the cell proliferation, clone formation, sphere formation, invasion and migration of hepatoma cells in vitro; conversely, these biological abilities of hepatoma cells were enhanced by HOXB7 overexpression. Moreover, the cancer stem cell markers EPCAM and NANOG were up-regulated by HOXB7. The role of HOXB7 in promoting tumor growth and metastasis was verified in vivo. Further investigation revealed that c-Myc and Slug expression was elevated by HOXB7 and the AKT pathway was activated.

**Conclusion:**

Overexpression of HOXB7 was significantly correlated with poor prognosis of HCC. HOXB7 up-regulated c-Myc and Slug expression via the AKT pathway to promote the acquisition of stem-like properties and facilitate epithelial-mesenchymal transition of hepatoma cells, accelerating the malignant progression of HCC.

## Background

Liver cancer is the second leading cause of cancer-related death worldwide, with an estimated 782 500 new cases and 745 500 deaths in 2012 [1]. As the major histological subtype, hepatocellular carcinoma (HCC) accounts for 70 to 85% liver cancers cases [2]. Recurrence and metastasis are frequently observed after surgery and mainly account for the poor prognosis of HCC [3]. Thus, a better understanding of the molecular mechanisms of HCC progression is urgently needed to facilitate the exploration of new therapeutic targets and the development of effective treatment strategies.

Homeobox genes encode a transcriptional family that contain 39 members classified into four groups (A, B, C, D), and these genes are essential for morphogenesis and differentiation [4]. Aberrant expression of homeobox genes is frequently detected in a variety of cancers [5]. Homeobox B7 (HOXB7), a member of homeobox gene family, has been shown to be involved in several cancers, including gastric [6], oral [7], pancreatic [8] and breast cancer [9]. As an important transcription factor, HOXB7 regulates many cancer cell functions, including proliferation, invasion, migration, angiogenesis and epithelial-mesenchymal transition (EMT) [10]. It has been reported that the proliferation and self-renewal of haematopietic stem cells are enhanced by HOXB7 [11]. In addition, HOXB7 facilitated the migration of breast cancer cells by inducing EMT [12]. As a fundamental process of migration, EMT plays a crucial role in HCC progression [13–15]. Moreover, overexpression of HOXB7 is significantly correlated with cancer progression and poor prognosis [16]. Recently, it has been reported that HOXB7 could modulate HCC cells proliferation and migration, and was significantly correlated with poor prognosis of HCC patients [17, 18]. However, the specific mechanism by which HOXB7 regulates HCC progression remains to be explored.

In this study, we aimed to analyse the potential mechanism of HOXB7 in HCC progression. First, we confirmed HOXB7 overexpression in HCC tissues, which is associated with poor outcomes of HCC patients. Furthermore, we investigated the role of HOXB7 in several cancer-related processes, including proliferation, stemness, invasion and migration of hepatoma cells in vitro and vivo. In addition, the specific mechanism by which HOXB7 promotes HCC progression was analysed.

## Methods

### Tissue samples from HCC patients

Seventy-seven preexisting paraffin-embedded tissue samples of HCC were collected from patients who underwent curative resection at the Institution of Hepatobiliary Surgery, Southwest Hospital, Third Military Medical University (Chongqing, China), from January 2010 to September 2015. All of the HCC tissue samples have paired adjacent noncancerous tissues. All patients have completed the follow-up information. The median follow-up time was 14 months, and the longest was 72 months. Clinical features of HCC patients were summarized in Table 1. All patients were informed with consent according to protocols approved by the Institutional Review Board of the Southwest Hospital, Third Military Medical University, and this study complied with by the ethical guidelines of the Helsinki Declaration.

**Table 1:** The relationship between expression of HOXB7 and clinical characteristics of 77 HCC patients

Variable	Number	Expression of HOXB7		*P* value
		Low level	High level	
Age (years)				
< 50	45	24	21	0.777
≥ 50	32	16	16	
Gender				
Female	5	2	3	0.586
Male	72	38	34	
Serum AFP level (ng/mL)				
< 400	35	18	17	0.935
≥ 400	42	22	20	
Tumor size(cm)				
< 5	32	21	11	0.043*
≥ 5	45	19	26	
Number of tumor				
Single	17	12	5	0.083
Multiple	60	28	32	
Differentiation				
High	21	15	6	0.008**
Moderate	36	19	17	
Low	20	6	14	
Vascular invasion				
-	32	22	10	0.012*
+	45	18	27	
Tumor stage (TNM)				
I/II	37	24	13	0.029*
III/IV	40	16	24	

* *P* < 0.05, **P < 0.01 significant difference

### Cell culture

The human hepatoma cells of HepG2 and Huh7 were purchased from American Type Culture Collection (Rockefeller, MD, USA); SMMC-7721, MHCC-97H, PLC and HEK-293T were purchased from the Cell Bank of Shanghai Institute of Cell Biology (Shanghai, China). MK-2206 was purchase from Selleck Chemicals. All cells were cultured in Dulbecco’s Modified Eagle Medium (DMEM) (Gibco, Grand Island, NY, USA) supplemented with 10% fetal bovine serum (FBS) (Gibco) at 37 °C in a humidified incubator containing 5% CO_2_.

### Lentivirus production and cell transduction

The lentivirus-luciferase-cherry plasmid was gifted by Professor Qian from the Center of Biotherapy, Southwest Hospital, Third Military Medical University. Lentivirus was produced by transfecting the packaging plasmids pMD2G, pRSV-Rev, and pMDLg/pRRE as well as the transfer lentiviral plasmids into HEK-293T cells using the calcium phosphate precipitation method. Lentivirus-containing supernatants were collected at 48 and 72 h, filtered through a 0.45-μm filter, and frozen at −80 °C until used to infect cells.

For shRNA or gene-encoded lentivirus was used to knockdown or overexpression HOXB7, cells were infected with the same virus MOI. After an overnight incubation with polybrene (2 μg/mL), the medium was replaced the following day. Protein expression was analysed by immunoblotting after 72 h of infection.

### Knockdown using shRNAs

shRNAs targeting HOXB7 were designed using RNAi software (Mekentosj), and three shRNAs targeting the coding sequence (CDS) of HOXB7 mRNA were inserted into the pLKO.1 vector (Addgene, plasmid 10878). The efficacy of each shRNA was assessed by western blot analysis of the endogenous protein in cells that had been infected with the viruses upon plating and cultured for 3 days. The shRNA with the strongest knockdown efficiency was selected for further experiments. The oligonucleotide sequences of the shRNAs were as follows:

- Scramble shRNA: 5’-CGTACGCGGAATACTTCGA-3’; HOXB7shRNA#1:5’-CGGAGCCTTCCCAGAACAAACTTCT-3’; HOXB7shRNA#2:5’-GAGCCGAGTTCCTTCAACATGCACT-3’; HOXB7shRNA#3:5’-GAACAAACTTCTTGTGCGTTTGCTT-3’

### Tumor growth in nude mice

The study was approved by the Animal Research Ethics Committee of Third Military Medical University, and complied with the Guidelines for Animal Experiments of Laboratory Animals. Eighteen 6-week-old mice were housed under specific pathogen-free conditions. SMMC-7721/scramble, SMMC-7721/shHOXB7, HepG2/vector and HepG2/HOXB7 (1 × 10^5^) cells were subcutaneously injected into the mice. Seven weeks after tumor began to form, the mice were sacrificed. Tumor growth was monitored by measuring the tumor diameter each week, and tumor weights were calculated at the end of the study. Volume of tumor = length × width^2^/2. After the mice sacrificed, tumors were taken out and fixed in OCT compound at −80 °C for further analysis.

### Metastasis model

Twenty-four 6-week-old nude mice aged were housed under specific pathogen-free conditions. SMMC-7721/scramble, SMMC-7721/shHOXB7, HepG2/vector and HepG2/HOXB7 cells were infected with lentivirus containing a luciferase-cherry gene, and cells expression cherry were selected by flow cytometry. SMMC-7721-Luc/scramble, SMMC-7721-Luc/shHOXB7, HepG2-Luc/vector and HepG2-Luc/HOXB7 cells (1 × 10^6^) were injected into the tail veins of mice to evaluate the effect of HOXB7 on the metastasis ability of HCC cells. An IVIS SPECTRUM system was used to observe the quantity and location of metastatic tumors in live mice 4 weeks after the cells were injected. After an intraperitoneal injection of luciferin (150 mg/kg), the mice were anaesthetized with 2% isoflurane. The bioluminescence of metastatic tumors was detected within 10 min. Tissue-specific metastasis was inspected immediately after sacrifice. After the mice were sacrificed, the tumors were removed and fixed in OCT compound at −80 °C for further analysis. This study was approved by the Animal Research Ethics Committee of Third Military Medical University, and complied with the Guidelines for Animal Experiments of Laboratory Animals.

### Histology and immunohistochemistry

Paraffin-embedded tissue samples from HCC patients were cut into 4-μm thick sections, deparaffinized with xylene, and rehydrated through graded alcohol washes.

Tumor tissues from mice were also sectioned at 4 μm thickness using a freezing microtome (Leica, Barnack, Germany). Antigen retrieval was performed for all sections by heating in a microwave oven, and endogenous peroxidase activity was blocked with 3% H_2_O_2_ solution. The sections were then incubated with an anti-HOXB7 antibody (1:500; Abcam, Cambridge, MA, USA) or an anti-Ki67 antibody (1:500; Abcam) at 4 °C overnight. In addition, lung sections from mice in the metastasis experiment were stained with haematoxylin and eosin (H&E). Immunohistochemistry assays were carried out using a DAKO EnVision Detection System.

Immunohistochemistry staining scores of HOXB7 in liver tissues were assessed using a semi-quantitative method by two pathologists who did not have knowledge of the clinical data as described previously [19]. The staining score was assessed as 0 (negative), 1 (weak), 2 (moderate) and 3 (strong). High expression was defined as a staining score of ≥ 2 with at least 50% of malignant cells showing positive HOXB7 staining, and a low expression level was defined as < 50% of malignant cells showing nuclear staining or a staining score < 2.

### Cell Titer-Blue cell proliferation assay

The assay was performed following the manufacturer’s instruction. Briefly, hepatoma cells (300 cells/well) transfected with sh-HOXB7/ad-HOXB7 and control cells were seeded into a 96-well culture plate. After 24 h incubation, 100 μL of DMEM containing 10 μL of Cell Titer-Blue® (Promega, Madison, WI, USA) was added to each well. The assay plates were incubated under standard cell culture conditions for 2 h, and the fluorescence was recorded at 560 nm using a spectrophotometer (Varioskan Flash, Thermo). The optical density (OD) was detected every 24 h for a total of 7 days. The experiment was repeated three times, and the mean values from triplicate wells were calculated.

### Clone formation assay

Briefly, SMMC-7721/scramble, SMMC-7721/shHOXB7, HepG2/vector and HepG2/HOXB7 cells were seeded in a 24-well plate at a density of 50 cells/well. The medium was replaced every 3 days and the cells were cultured for 14 days. The clones were fixed with 4% formaldehyde for 10 min and stained with 0.5% crystal violet for 3 min (Sigma-Aldrich). A clone was defined as > 50 cells. Clone formation rate (%) = (number of clones/number of seeded cells) × 100%.

### Sphere formation assay

The sphere formation assay was performed as described previously [19]. SMMC-7721/scramble, SMMC-7721/shHOXB7, HepG2/vector and HepG2/HOXB7 cells were seeded into low-attachment 96-well plates at density of 10 cells/well. The cells were cultured in DMEM/F12 medium (Sigma) mixed with B27 supplement (Gibco), antibiotics, 20 ng/mL epidermal growth factor, 20 ng/mL basic fibroblast growth factor (Peprotech) and 10 ng/mL hepatocyte growth factor (Peprotech), and 1% methylcellulose was added to prevent cell aggregation. The cells were incubated at 37 °C for 14 days, and the medium was added every 3 days. Sphere with a diameter > 75 μm were counted. Sphere formation rate (100%) = (number of spheres/number of seeded cells) × 100%.

### Immunofluorescence

Hepatoma cells were seeded onto glass coverslips placed in 24-well plates and then cultured at 37 °C in a humidified incubator with 5% CO_2_. After culturing for 48 h, the cells were fixed with 500 μl of 4% formaldehyde for 10 min at room temperature. The coverslips were then washed with PBS three times, and nonspecific binding sites were blocked with a solution of 1% bovine serum albumin in PBS for 30 min. A mouse monoclonal anti-HOXB7 antibody (Abcam) (1:250) was added to the cells and incubated overnight at 4 °C. The cells were then incubated with an Alexa Fluor® 647-conjugated secondary antibody (1:1000) for 30 min at 37 °C in the dark, and the cell nuclei were stained with 2-(4-amidinophenyl)-6-indolecarbamidine dihydrochloride (DAPI) (Sigma-Aldrich). Finally, a confocal laser-scanning microscope (LSM 780, Carl Zeiss, Germany) was used to detect the location of HOXB7.

### Cell migration and invasion assay

A 24-well plate with 8-μm pore size polycarbonate membrane inserts (Millipore, Billerica, MA, USA) was used to evaluate cell motility. For the migration assay, cells (8.0 × 10^4^) were seeded in the upper chamber in 300 μL of serum-free DMEM, and 800 μl of DMEM with 20% serum was added to the lower chamber. For the invasion assay, the membranes were used to pre-coat with Matrigel (2 μg/well; BD Biosciences, San Jose, CA, USA) to simulate a matrix barrier. Hepatoma cells (8.0 × 10^4^) were suspended in 300 μL serum-free DMEM medium and added to the upper part of the insert chamber, and 800 μL of DMEM with 20% serum was added to the lower chamber. After culturing for 24 h for the migration assay and 48 h for the invasion assay, non-migrated cells were removed from the upper chamber. Cells that passed through the membrane were fixed with 4% formaldehyde and stained with 0.1% crystal violet. Five visual fields were randomly selected from each membrane, and the cell numbers were counted via a light microscope at 100 × magnification. All experiments were performed in triplicate.

### Wound-healing assay

HepG2 and SMMC-7721 cells were plated in a 24-well plate and grown to confluence. Then, the cell layers were scratched for the wound-healing assay. A linear wound was created by dragging a 1-μl pipette tip through the monolayer. The hepatoma cells were washed with PBS, and then complete medium was added. Photographs were taken at 100 × magnification at 0, 18 and 36 h post-wounding, and the cells were evaluated under an Olympus inverted microscope.

### Western blot analysis

Total protein was extracted from tumor samples or cells. The protein samples were separated by SDS-PAGE and transferred to nitrocellulose membranes. After blocking via 5% skimmed milk, membranes were incubated (overnight, 4°C) with specific primary antibodies as follows: anti-HOXB7 antibody, anti-NANOG antibody, anti-EPCAM antibody, anti-ERK antibody, anti-phosphorylated ERK antibody, anti-c-Myc antibody, anti-E-cadherin antibody, anti-N-cadherin antibody, anti-Slug antibody, anti-Vimentin antibody, anti-α-SMA antibody (Abcam); anti-MMP2 antibody, anti-MMP9 antibody, anti-GAPDH antibody, anti-SMAD3 antibody, anti-phosphorylated SMAD3, anti-p38 antibody, anti-phosphorylated p38, anti-AKT antibody, anti-phosphorylated AKT antibody (Cell Signaling Technology, Danvers, MA, USA). After washing, membranes were incubated 2 h with secondary antibodies (Sigma-Aldrich). The protein intensity was determined and measured by Image Lab software (5.2.1 Version, Bio-Rad Laboratories Co. Ltd, CA, USA). After normalization to GAPDH protein units for each sample, the semi-quantitate results for either tumor or adjacent samples were obtained as a ratio.

### RNA isolation and real-time quantitative reverse transcription PCR

According to the manufacture’s instruction, total RNA of HepG2 and SMMC-7721 were extracted and purified by Trizol (Takara, Japan). Then cDNA was synthesized using 1 μg of RNA via reverse transcriptional kit abide by manufacture’s protocol. Quantitative PCR was performed by SYBR premix ExTaq (Takara) on CFX96 Real Time PCR Detection System (Bio-Rad). GAPDH as an internal control was used to normalize the mRNA expression of each gene. All reactions were detected in triplicate, at least three independent times for each experiment was performed. Related primer sequences were listed in Table 2.

**Table 2:** Primers have been used for real time PCR

Primer name	Sequence 5’-3’	Product length (bp)
HOXB7	Forward:CGAGTTCCTTCAACATGCACT	170 bp
	Reverse:TTTGCGGTCAGTTCCTGAGC	
c-Myc	Forward:CGTCCTCGGATTCTCTGCTC	280 bp
	Reverse:GCTGCGTAGTTGTGCTGATG	
Sox2	Forward:TACAGCATGTCCTACTCGCAG	110 bp
	Reverse:GAGGAAGAGGTAACCACAGGG	
Sox9	Forward:AAGAACAAGCCGCACGTCAA	257 bp
	Reverse:CCGTTCTTCACCGACTTCCTC	
Oct4	Forward:CAAAGCAGAAACCCTCGTGC	171 bp
	Reverse:AACCACACTCGGACCACATC	
Twist1	Forward:ATTCAAAGAAACAGGGCGTGG	103 bp
	Reverse:CAGAGGTGTGAGGATGGTGC	
Slug	Forward:TGTGACAAGGAATATGTGAGCC	203 bp
	Reverse:TGAGCCCTCAGATTTGACCTG	
GAPDH	Forward:TGCCACTCAGAAGACTGTGG Reverse:TTCAGCTCTGGGATGACCTT	129 bp

### Statistical analysis

The data were expressed as the mean ± standard deviation (SD) from a minimum of three separate experiments. The significance of differences was examined using Student’s *t*-test. The correlations of clinical characteristics in HCC patients were analysed using the chi-squared test. Survival data were used to draw Kaplan-Meier curves, and the differences among the groups were analysed by log-rank assay. Statistical analyses were performed using SPSS 21.0 (SPSS Inc., Chicago, IL, USA). *P* < 0.05 was considered to indicate a statistically significant difference.

## Results

### HOXB7 expression is up-regulated in human HCC tissues and correlated with poor prognosis of HCC

**Fig. 1:**
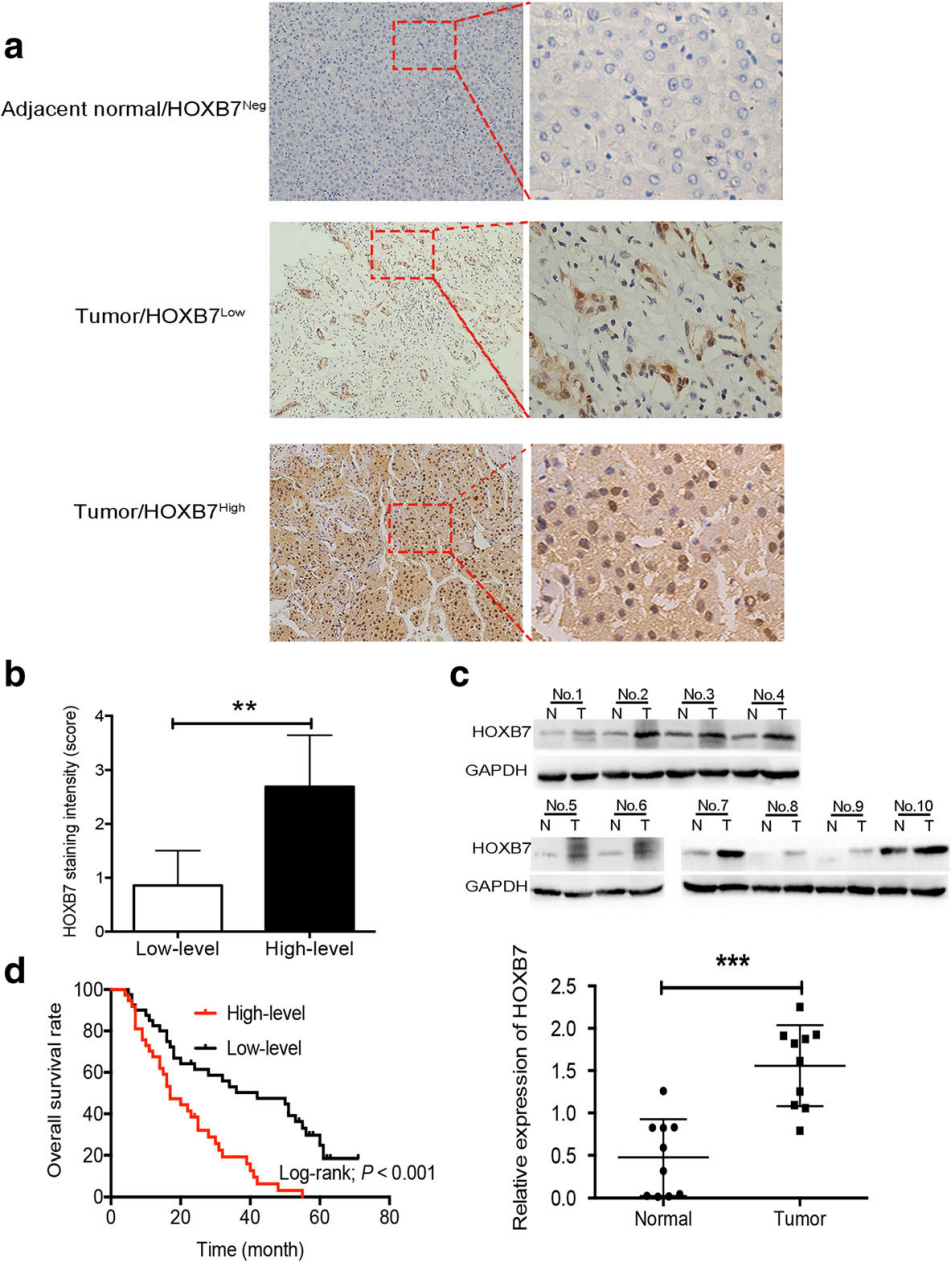
Up-regulation of HOXB7 in HCC tissues correlates with poor prognosis of HCC. **a** A representative image showing the expression level of HOXB7 in HCC and paired adjacent noncancerous tissues. HOXB7 was negative in adjacent noncancerous tissue and positive in HCC tissue. **b** IHC staining scores were used to define low and high expression of HOXB7 in HCC tissues (n = 77; *P* < 0.01). **c** *Upper panel*: protein level of HOXB7 in 10 randomly selected paired HCC tissues was determined by western blot. *Lower panel*: semi-quantitative analysis of the western blot results. GAPDH was used to normalize the expression level of HOXB7 (n = 10; *P* < 0.001). **d** Kaplan-Meier survival analysis of HCC patients. A high level of HOXB7 resulted in a shorter survival time in HCC patients (n = 37); a low level of HOXB7 was correlated with a longer survival time (n = 40; *P* < 0.001)

To examine the correlation between HOXB7 and HCC prognosis, the expression of HOXB7 in 77 HCC specimens and corresponding paired adjacent noncancerous tissues was detected. Our results showed that the expression level of HOXB7 was higher in HCC tissues than in paired noncancerous tissue, and there was little to no staining of HOXB7 in non-cancerous tissue. Moreover, the expression level of HOXB7 varied in the HCC tumor tissues (Fig. 1a). IHC staining scores also demonstrated the variable expression level of HOXB7 in HCC tissues and were used to define high and low expression of HOXB7 in HCC tissues (Fig. 1b). A high expression level was defined as a staining score of ≥ 2 with at least 50% of the malignant cells exhibiting positive HOXB7 staining, and a low expression level was defined as a staining score < 2 or < 50% of the malignant cells showing nuclear staining. Further exploration revealed that the protein level of HOXB7 was up-regulated in 10 randomly selected paired specimens analysed via western blot (*P* < 0.001; Fig. 1c).

Statistical analysis showed that higher expression of HOXB7 in HCC tissue was positively associated with tumor size (*P* = 0.043), differentiation degree (*P* = 0.008), vascular invasion (*P* = 0.012) and TNM stage of HCC (*P* = 0.029; Table 1). However, there was no significant relationship between the degree of HOXB7 staining and age or the number of tumor (Table 1). Moreover, our results suggested that a higher expression level of HOXB7 in HCC tissue was correlated with a shorter overall survival time (*P* < 0.001; Fig. 1d). In summary, our data certified that aberrant overexpression of HOXB7 in HCC tissue was significantly correlated with poor prognosis of HCC.

### HOXB7 facilitated hepatoma cell proliferation by promoting stemness

**Fig. 2:**
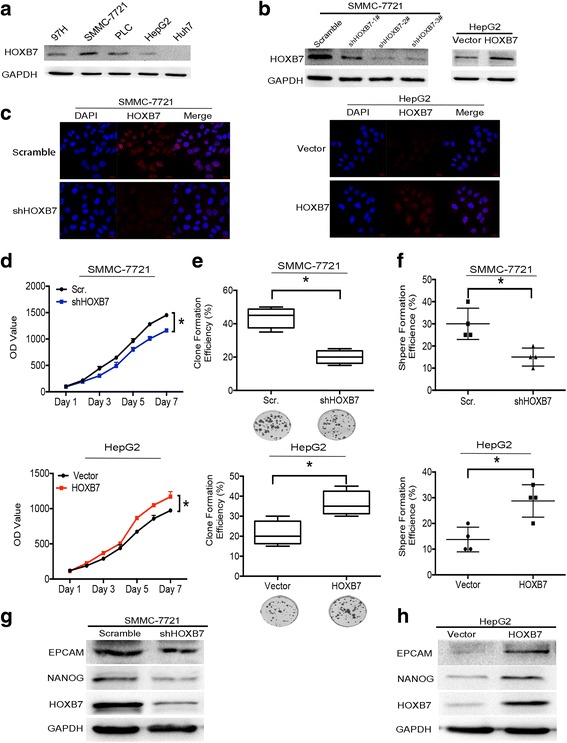
HOXB7 facilitated hepatoma cell proliferation by promoting stemness. **a** Protein level of HOXB7 in different hepatoma cell lines. **b** Efficiency of knockdown and overexpression HOXB7 in SMMC-7721 and HepG2 cells, respectively. **c** Location of HOXB7 in SMMC-7721 and HepG2 cells. **d** Cell Titer-Blue assay was used to examine the effect of HOXB7 on the proliferation of SMMC-7721 and HepG2 cells. HOXB7 promoted the proliferation of hepatoma cells (*P* < 0.05, respectively). **e** Analysis of clone formation in SMMC-7721 and HepG2 cells. HOXB7 promoted clone formation of hepatoma cells (*P* < 0.05, respectively). **f** Analysis of sphere formation in SMMC-7721 and HepG2 cells. HOXB7 accelerated sphere formation of hepatoma cells (*P* < 0.05, respectively). **g**, **h** Hepatoma stem cell markers EPCAM and NANOG expression level after HOXB7 knockdown and overexpression

After verifying the association between HOXB7 and HCC prognosis, we next sought to identify the biological function of HOXB7 in hepatoma cell growth. The expression level of HOXB7 in different human hepatoma cell lines was detected, and SMMC-7721 cells exhibited higher expression level of HOXB7, while the expression in HepG2 was lower (Fig. 2a). Thus, we used SMMC-7721 and HepG2 cells to evaluate the biological function of HOXB7 in HCC process. To assess the impact of HOXB7 on growth of hepatoma cells, we knocked down HOXB7 in SMMC-7721 cells using an shRNA specific to HOXB7 (HOXB7 shRNA#2) and overexpressed HOXB7 in HepG2 cells (Fig. 2b). HOXB7 was located in the nucleus of both HepG2 and SMMC-7721 cells (Fig. 2c). Knockdown of HOXB7 inhibited SMMC-7721 cells proliferation (*P* < 0.05; Fig. 2d). Conversely, proliferation of HepG2 cells was promoted by HOXB7 overexpression (*P* < 0.05; Fig. 2d). Further investigation showed that knockdown of HOXB7 in SMMC-7721 cells inhibited clone formation and sphere formation (*P* < 0.05; Fig. 2e, f), while HepG2 cells exhibited higher clone formation and sphere formation rates after overexpression of HOXB7 (*P* < 0.05; Fig. 2e, f). These results confirmed that HOXB7 accelerated hepatoma cell growth in vitro.

Proliferation, sphere formation and clone formation are characteristics of cancer stem cells. To determine whether HOXB7 regulates the stemness of hepatoma cells, we analysed the expression of cancer stem cell markers. Knockdown of HOXB7 in SMMC-7721 cells inhibited EPCAM and NANOG expression (Fig. 2g), while overexpression of HOXB7 in HepG2 cells up-regulated EPCAM and NANOG (Fig. 2h). These results suggested that HOXB7 could regulate the proliferation of hepatoma cells by modulating stem-related characteristics.

### HOXB7 promoted EMT to enhance the migration and invasion of hepatoma cells

**Fig. 3:**
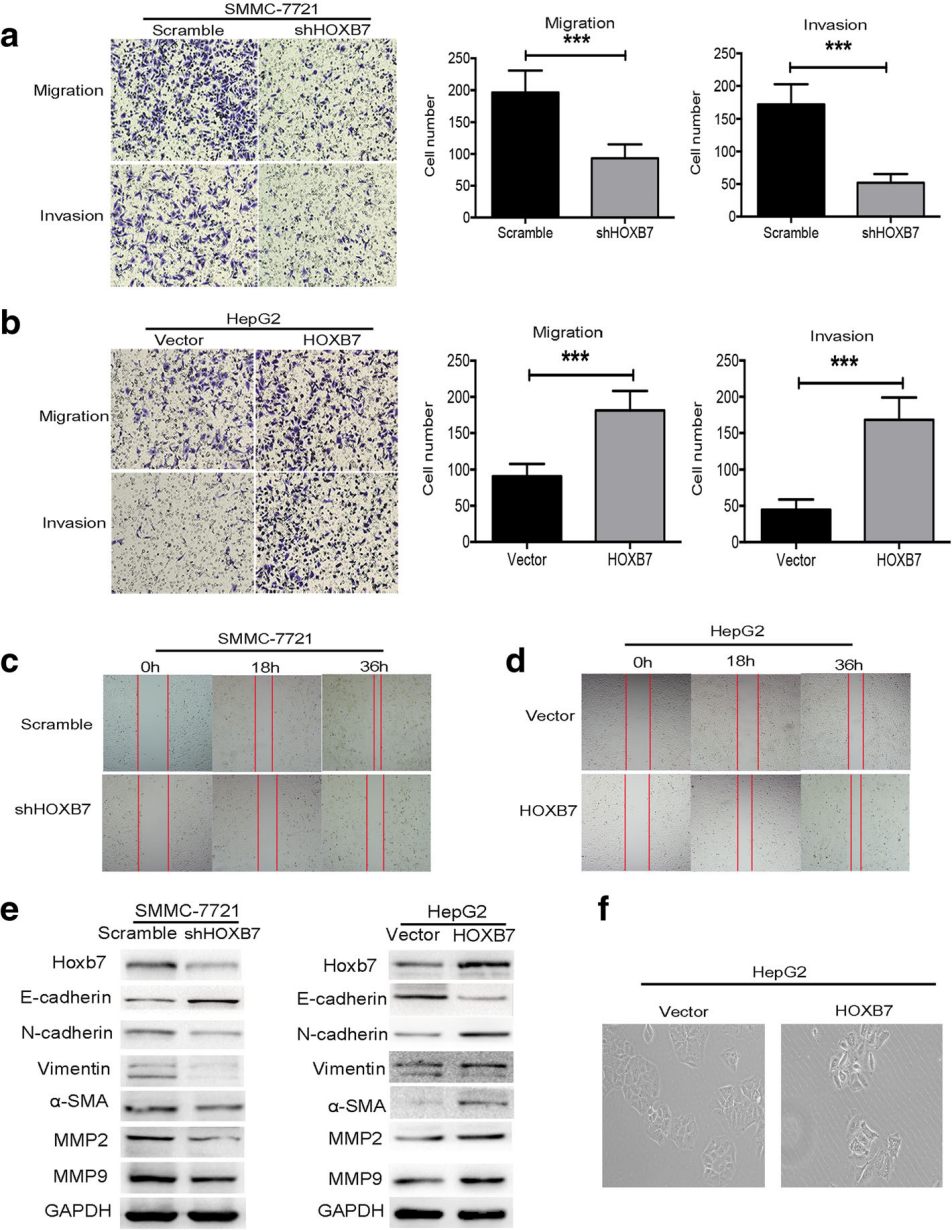
HOXB7 promoted EMT to enhance the migration and invasion of hepatoma cells. **a** Knockdown of HOXB7 inhibited the migration and invasion of SMMC-7721 cells; cell counting showed that invasion and migration were evidently inhibited by knockdown of HOXB7 in SMMC-7721 cells (*P* < 0.001, respectively). **b** Overexpression of HOXB7 facilitated HepG2 cell migration and invasion (*P* < 0.001, respectively). All images were taken under × 100 magnification. **c**, **d** Wound healing assay was used to examine the effect of HOXB7 on SMMC-7721 and HepG2 cells migration. Both figures revealed that HOXB7 promoted hepatoma cells migration. **e** HOXB7 reduced the expression of E-cadherin and up-regulated the expression of N-cadherin, Vimentin, α-SMA, MMP2 and MMP9 in SMMC-7721 and HepG2 cells, respectively. **f** Changes of cell morphology after overexpression of HOXB7

Metastasis is also vital for cancers progression. HOXB7 has been reported to regulate the invasion and migration of cancer cells. The effect of HOXB7 on the invasion and migration abilities of hepatoma cells was examined. Transwell assays showed that knockdown of HOXB7 weakened the invasion and migration abilities of SMMC-7721 cells (*P* < 0.001, respectively; Fig. 3a). Conversely, these abilities were enhanced after overexpression of HOXB7 in HepG2 cells (*P* < 0.001, respectively; Fig. 3b). Furthermore, wound-healing assay showed that knockdown of HOXB7 inhibited the migration of SMMC-7721 cells, while the migration ability of HepG2 cells was enhanced by overexpression of HOXB7 (Fig. 3c, d). To further validate the relationship between HOXB7 and HCC metastasis, we detected the protein level of N-cadherin, E-cadherin, Vimentin, α-SMA, matrix metalloproteinase-2 (MMP2) and matrix metalloproteinase-9 (MMP9). We found that HOXB7 increased the expression of N-cadherin, Vimentin, α-SMA, MMP2 and MMP9, and reduced the expression of E-cadherin (Fig. 3e). Moreover, overexpression of HOXB7 promoted HepG2 cells to switch to the spindle-like form (Fig. 3f). Our results implied that HOXB7 had a strong effect on the EMT phenotypes of hepatoma cells, and it regulated the malignant progression of HCC by promoting EMT.

### HOXB7 promoted tumor growth in vivo

**Fig. 4:**
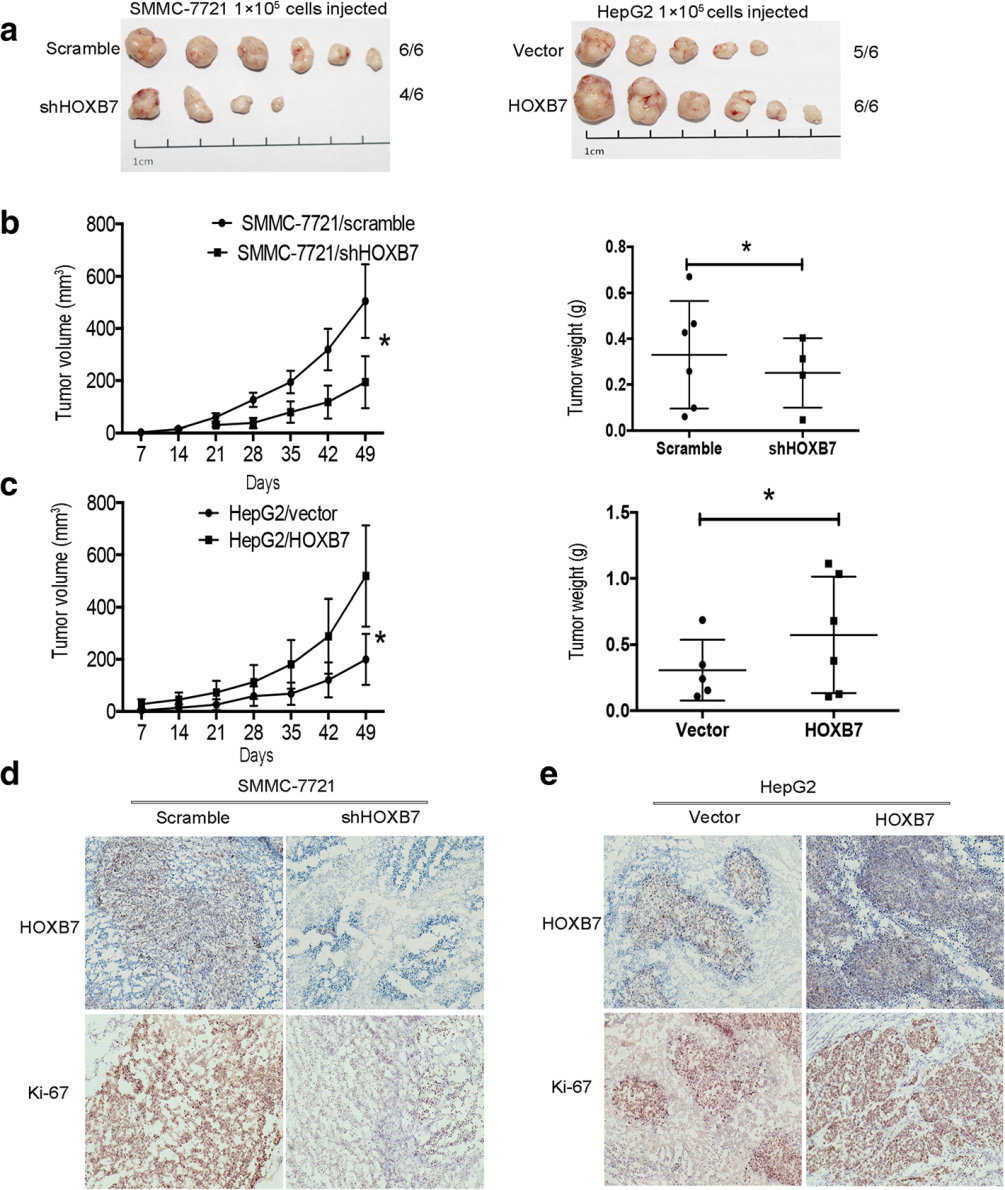
Overexpression of HOXB7 facilitated tumor growth in vivo. **a** SMMC-7721/scramble, SMMC-7721/shHOXB7, HepG2/vector and HepG2/HOXB7 cells were subcutaneously injected in the hind limbs of nude mice (*n* = 6/group; 1 × 10^5^ cells). **b**, **c** Tumor weight and volume were measured in each group (*P* < 0.05, respectively). **d**, **e** Representative histopathology of xenograft tumors. The tumor sections were subjected to IHC staining using antibodies against HOXB7 and Ki-67

To determine whether HOXB7 promote tumor growth in vivo, a xenograft tumor model was established. The number of tumors was reduced after HOXB7 knockdown in SMMC-7721 cells, while more and bigger tumors formed after HOXB7 overexpression in HepG2 cells (Fig. 4a). Further assessment revealed that HOXB7 expression promoted tumor growth and resulted in larger tumors (*P* < 0.05; Fig. 4b, c). In addition, HOXB7 knockdown in SMMC-7721 cells was accompanied by light staining of Ki-67 in tumors (Fig. 4d), and the staining of Ki-67 was enhanced following overexpression of HOXB7 in HepG2 cells (Fig. 4e). Collectively, these data showed that HOXB7 was essential for HCC growth.

### HOXB7 facilitated metastasis of hepatoma cells in vivo

**Fig. 5:**
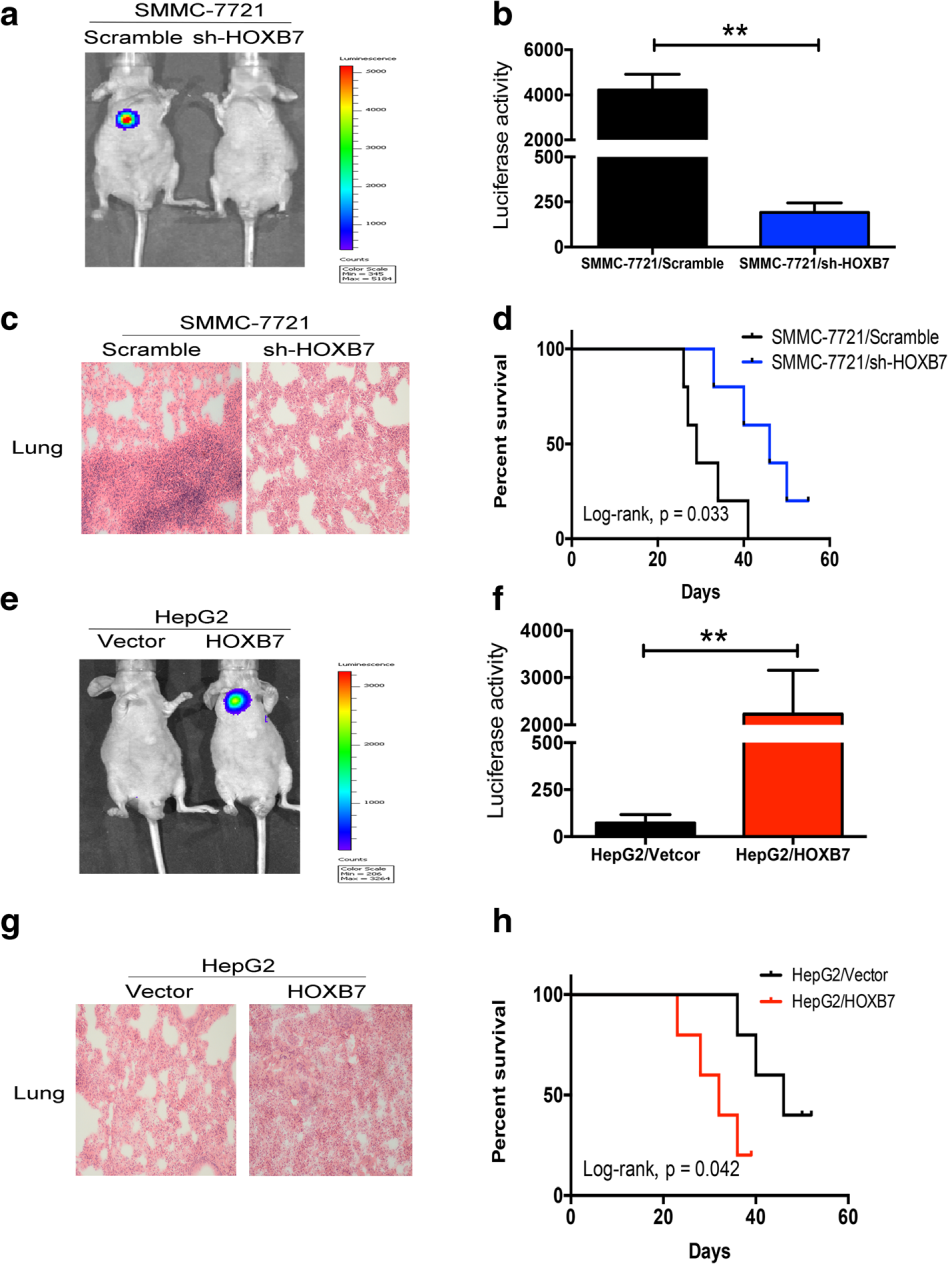
Effect of HOXB7 on metastasis of hepatoma cells in vivo. **a**, **e** Representative images of metastasis to the lung in the SMMC-7721/scramble, SMMC-7721/shHOXB7, HepG2/vector and HepG2/HOXB7 groups (*n* = 6 per group). **b**, **f** Quantification of fluorescence from metastatic tumors (*P* < 0.01, respectively). **c**, **g** H&E staining of metastatic tumors in lung tissues. **d**, **h** Kaplan-Meier survival analysis of mice in different groups

To further validate the role of HOXB7 in HCC metastasis, we established a metastasis model in nude mice. Knockdown of HOXB7 in SMMC-7721 cells inhibited metastasis, and SMMC-7721/scramble cells expressing more HOXB7 formed a conspicuous metastatic tumor in the lung (Fig. 5a). Similarly, overexpression of HOXB7 in HepG2 cells promoted metastasis and colonization in the lung (Fig. 5e). Quantification of luciferase activity showed that HOXB7 conferred stronger metastasis ability on hepatoma cells (*P* < 0.01; Fig. 5b, f). H&E staining of metastatic tissues demonstrated the colonization of HCC cells in the lung. It was easier for HCC cells expressed higher level of HOXB7 to colonize the lung (Fig. 5c, g). Survival analysis revealed that HOXB7 knockdown in hepatoma cells contributed to a longer survival time and up-regulation of HOXB7 led to a worse outcome (Fig. 5d, h). Thus, HOXB7 accelerated HCC metastasis.

### HOXB7 up-regulated c-Myc and Slug expression via the AKT pathway to promote HCC progression

**Fig. 6:**
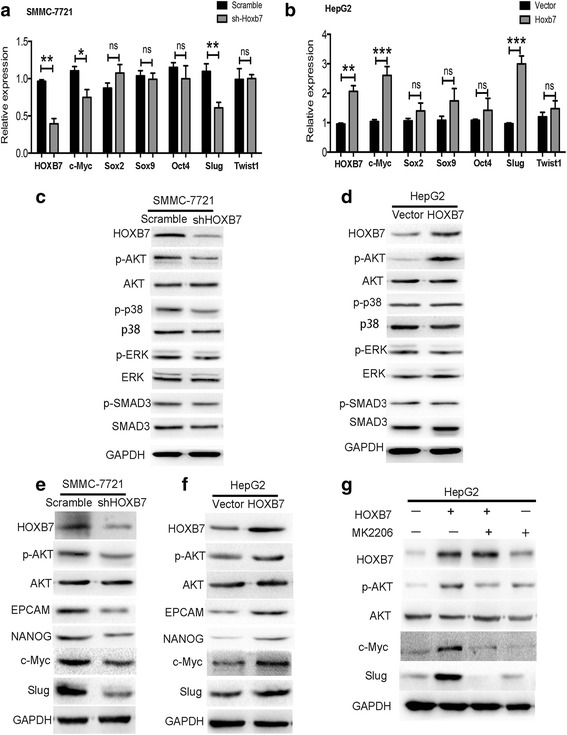
HOXB7 up-regulated c-Myc and Slug expression via the AKT pathway to promote HCC progression. **a**, **b** RT-PCR was used to investigate the effect of HOXB7 on stemness and metastasis related genes such as Sox2, Sox9, c-Myc, Oct4, Slug and Twist1 in SMMC-7721 and HepG2 cells (*, *P* < 0.05; **, *P* < 0.01; ***, *P* < 0.001). **c**, **d** The expression of AKT, p-AKT, ERK, p-ERK, p-38, p-p38, SMAD3 and p-SMAD3 in hepatoma cells were analysed by western blot. **e**, **f** HOXB7 up-regulated EPCAM, NANOG, Slug, c-Myc and promoted phosphorylation of AKT. **g** Pharmacologically inhibiting AKT depressed the effect of HOXB7 on c-Myc and Slug expression

The results above verified that dysregulation of HOXB7 is significantly associated with the malignant progression of HCC. However, the exact mechanism remains unclear. To elucidate the mechanism by which HOXB7 regulates HCC progression, several genes related to stemness and metastasis were analysed after HOXB7 knockdown or overexpression, including Sox2, Sox9, c-Myc, Oct4, Slug, and Twist1. Knockdown HOXB7 in SMMC-7721 cells inhibited c-Myc and Slug at transcription level, and overexpression of HOXB7 up-regulated c-Myc and Slug in HepG2 cells (Fig. 6a, b). Moreover, we screened related signaling pathways, including AKT, p-38, SMAD3 and ERK, which have been demonstrated to be modulated by HOXB7. Knockdown of HOXB7 in SMMC-7721 cells suppressed the phosphorylation of AKT, and p-ERK, p-SMAD3, p-p38 were also slightly depressed (Fig. 6c). Conversely, overexpression of HOXB7 strongly increased p-AKT expression in HepG2 cells, but had little effect on other pathways (Fig. 6d). Moreover, the protein levels of c-Myc and Slug were up-regulated by HOXB7, accompanied with increasingly phosphorylation of AKT, and EPCAM or NANOG were also up-regulated (Fig. 6e, f). Our data suggested that HOXB7 accelerated cell growth and metastasis of HCC through activated AKT pathway to up-regulate c-Myc and Slug.

To verify that the AKT pathway was the main pathway participating in the regulation of c-Myc and Slug by HOXB7, MK2206 was used to inhibit AKT activity pharmacologically and then detected the changes of c-Myc and Slug expression after HOXB7 overexpression. Overexpression of HOXB7 strongly up-regulated c-Myc and Slug, but blocking AKT inhibited this effect (Fig. 6g). Thus, the AKT pathway is the main signalling pathway that participates in the regulation of HCC progression by HOXB7. Collectively, our data suggested that HOXB7 accelerated hepatoma cell growth and metastasis by activating the AKT pathway to up-regulate c-Myc and Slug.

## Discussion

HOXB7 is a member of the homeobox gene family and has been shown to play an important role in various cancer-related processes, including proliferation [7], metastasis [9], and angiogenesis [20]. Here, we showed that HOXB7 was over expression in HCC tissues and correlated with poor prognosis of HCC patients. Overexpression of HOXB7 in hepatoma cells enhanced growth and metastasis in vitro and vivo. Furthermore, HOXB7 facilitated the acquisition of stem-like properties and EMT. Overexpression of HOXB7 promoted the phosphorylation of AKT and up-regulated c-Myc and Slug. Our results suggested that HOXB7 promotes the stemness and EMT of hepatoma cells to accelerate the malignant progression of HCC by up-regulating c-Myc and Slug.

It is generally recognized that HOXB7 is dysregulated in several types of cancers and associated with cancer progression [10]. HOXB7 up-regulation has been demonstrated in gastric cancer and shown to indicate poor prognosis in gastric cancer patients [21]. Deregulation of HOXB7 expression in esophageal squamous cell carcinoma predicted poor outcomes of patients [22]. Up-regulation of HOXB7 in pancreatic ductal adenocarcinoma was correlated with advanced stage of the disease [23]. Recently, it has reported that HOXB7 could modulate hepatoma cells proliferation and migration, and that it was significantly correlated with poor prognosis of HCC [17, 18]. However, the specific mechanism by which HOXB7 regulates the malignant progression of HCC was unclear. Our study confirmed that HOXB7 was associated with poor prognosis of HCC. Moreover, our results revealed that HOXB7 enhanced cancer stem-like properties and EMT in hepatoma cells.

It has been reported that HOXB7 promotes haematopoietic stem cell self-renewal and proliferation [11], and HOXB7 is a master factor that drives the progenitor’s behaviour of mesenchymal stromal/stem cells [24]. In line with previous reports, our results showed that HOXB7 facilitated hepatoma cells proliferation. In addition, clone formation and sphere formation of hepatoma cells were accelerated and cancer stem markers including EPCAM and NANOG were up-regulated. These data indicated that HOXB7 facilitated HCC growth by regulating stemness of hepatoma cells.

A number of investigations have demonstrated that HOXB7 plays an important role in modulating cell invasion and migration. A recent study has reported that HOXB7 promoted the migration and invasion of hepatoma cells [18], but the related mechanism was not clear. EMT plays a fundamental role in HCC metastasis [13, 25], it causes epithelial cells to lose their cell-cell adhesions [26], undergo cytoskeleton remodeling [27] and acquire a more mesenchymal and invasive/metastatic phenotype [28]. HOXB7 induced EMT and promoted migration and invasion in breast cancer cells [12]. HOXB7 knockdown resulted in depression of migration and invasion with EMT alteration in lung adenocarcinoma [29]. Our results revealed that HOXB7 had a substantial impact on the EMT phenotypes of hepatoma cells, enhancing hepatoma cell motility and playing an important role in EMT.

Although the role of HOXB7 in progression of HCC has been verified, the specific mechanism of HOXB7 regulating HCC remains to be explored. Further investigation showed that the expression of c-Myc and Slug was up-regulated by HOXB7, indicating that HOXB7 promoted the malignant progression of HCC by up-regulating c-Myc and Slug. Meanwhile, the AKT pathway was activated by HOXB7 in hepatoma cells. A previous study reported that HOXB7 facilitated the proliferation of colorectal cancer cells by activating the PI3K/AKT pathway [30]. Moreover, the PI3K/AKT/c-Myc axis is crucial for esophageal squamous cancer cells to obtain cancer stem-like features [31]. Our results showed that the PI3K/AKT/c-Myc axis was modulated by HOXB7 to facilitate the acquisition of cancer stem-like properties in hepatoma cells. It has also been reported that gastric cancer cell metastasis is promoted by HOXB7 via the PI3K/AKT pathway [6, 32, 33]. The AKT pathway was involved in EMT and carcinogenesis via the up-regulation of Slug [34]. Our results revealed that HOXB7 facilitated hepatoma cell metastasis and up-regulated Slug expression. This suggested that HOXB7 promoted the metastasis of hepatoma cells by activating PI3K/AKT/Slug to promote EMT. In brief, HOXB7 promoted the malignant progression of HCC by activating AKT pathway to up-regulate c-Myc and Slug.

Several studies have demonstrated that the progression of several types of cancer is regulated by HOXB7 via AKT [30], ERK [18], TGF-β [35], Ras/Rho [12] and VEGF [36]. A recent study has also reported that HOXB7 accelerated hepatoma cells proliferation and metastasis by activating the MAPK/ERK pathway [18]. The authors of this study directly detected the impact of HOXB7 on the activation status of ERK. However, in our study, we screened several pathways related to HOXB7. We found that AKT phosphorylation was promoted by HOXB7, which indicating that the AKT pathway was activated. Furthermore, p-ERK, p-p38 and p-SMAD3 decreased after HOXB7 knockdown, but there were no significant changes after overexpression of HOXB7. The PI3K/AKT pathway has been demonstrated to participate in proliferation, EMT and stemness [37]. Our study verified that HOXB7 enhanced hepatoma cell metastasis by promoting EMT and facilitated HCC growth by promoting stemness. These results implied that HOXB7 could regulate several signaling pathways but that the AKT pathway was the primary pathway activated by HOXB7 to modulate the malignant progression of HCC.

## Conclusion

In conclusion, our study confirmed that HOXB7 overexpression was significantly correlated with poor prognosis of HCC patients. Further investigation identified that HOXB7 not only promoted EMT, but also facilitated the acquisition of cancer stem-like properties in hepatoma cells. Mechanistically, HOXB7 promoted c-Myc and Slug expression via the AKT pathway, which could be a valuable marker of HCC prognosis and a novel target for HCC treatment.

## Abbreviations

bFGF: Basic fibroblast growth factor
DMEM: Dulbecco’s Modified Eagle Medium
EGF: Epidermal growth factor
EMT: Epithelial-mesenchymal transition
FBS: Fetal bovine serum
H&E: Hematoxylin and Eosin
HCC: Hepatocellular carcinoma
HGF: Hepatocyte growth factor
HOXB7: Homeobox B7
IHC: Immunohistochemistry
OD: Optical density
SD: Standard deviation

## References

[1] Torre LA, Bray F, Siegel RL, Ferlay J, Lortet-Tieulent J, Jemal A (2015). Global cancer statistics, 2012. CA Cancer J Clin.

[2] Perz JF, Armstrong GL, Farrington LA, Hutin YJ, Bell BP (2006). The contributions of hepatitis B virus and hepatitis C virus infections to cirrhosis and primary liver cancer worldwide. J Hepatol.

[3] Forner A, Llovet JM, Bruix J (2012). Hepatocellular carcinoma. Lancet.

[4] Tschopp P, Duboule D (2011). A genetic approach to the transcriptional regulation of Hox gene clusters. Annu Rev Genet.

[5] Shah N, Sukumar S (2010). The Hox genes and their roles in oncogenesis. Nat Rev Cancer.

[6] Joo MK, Park JJ, Yoo HS, Lee BJ, Chun HJ, Lee SW (2016). The roles of HOXB7 in promoting migration, invasion, and anti-apoptosis in gastric cancer. J Gastroenterol Hepatol.

[7] De Souza Setubal Destro MF, Bitu CC, Zecchin KG, Graner E, Lopes MA, Kowalski LP (2010). Overexpression of HOXB7 homeobox gene in oral cancer induces cellular proliferation and is associated with poor prognosis. Int J Oncol.

[8] Chile T, Fortes MA, Correa-Giannella ML, Brentani HP, Maria DA, Puga RD (2013). HOXB7 mRNA is overexpressed in pancreatic ductal adenocarcinomas and its knockdown induces cell cycle arrest and apoptosis. BMC Cancer.

[9] Chen H, Lee JS, Liang X, Zhang H, Zhu T, Zhang Z (2008). HOXB7 inhibits transgenic HER-2/neu-induced mouse mammary tumor onset but promotes progression and lung metastasis. Cancer Res.

[10] Errico MC, Jin K, Sukumar S, Care A (2016). The Widening Sphere of Influence of HOXB7 in Solid Tumors. Cancer Res.

[11] Care A, Valtieri M, Mattia G, Meccia E, Masella B, Luchetti L (1999). Enforced expression of HOXB7 promotes hematopoietic stem cell proliferation and myeloid-restricted progenitor differentiation. Oncogene.

[12] Wu X, Chen H, Parker B, Rubin E, Zhu T, Lee JS (2006). HOXB7, a homeodomain protein, is overexpressed in breast cancer and confers epithelial-mesenchymal transition. Cancer Res.

[13] Yoshida GJ (2016). Emerging role of epithelial-mesenchymal transition in hepatic cancer. J Exp Clin Cancer Res.

[14] Huang D, Cao L, Zheng S (2017). CAPZA1 modulates EMT by regulating actin cytoskeleton remodelling in hepatocellular carcinoma. J Exp Clin Cancer Res.

[15] Zhang Y, Zeng S, Ma J, Deng G, Qu Y, Guo C (2016). Nestin overexpression in hepatocellular carcinoma associates with epithelial-mesenchymal transition and chemoresistance. J Exp Clin Cancer Res.

[16] Liu FT, Ou YX, Zhang GP, Qiu C, Luo HL, Zhu PQ (2016). HOXB7 as a promising molecular marker for metastasis in cancers: a meta-analysis. Onco Targets Ther.

[17] Komatsu H, Iguchi T, Masuda T, Ueda M, Kidogami S, Ogawa Y (2016). HOXB7 Expression is a Novel Biomarker for Long-term Prognosis After Resection of Hepatocellular Carcinoma. Anticancer Res.

[18] Wang WM, Xu Y, Wang YH, Sun HX, Sun YF, He YF, et al. HOXB7 promotes tumor progression via bFGF-induced activation of MAPK/ERK pathway and indicated poor prognosis in hepatocellular carcinoma. Oncotarget. 2017. doi:10.18632/oncotarget.17004. Epub ahead of print. 10.18632/oncotarget.17004 PMC5564549 28454092

[19] Shan J, Shen J, Liu L, Xia F, Xu C, Duan G (2012). Nanog regulates self-renewal of cancer stem cells through the insulin-like growth factor pathway in human hepatocellular carcinoma. Hepatology.

[20] Care A, Felicetti F, Meccia E, Bottero L, Parenza M, Stoppacciaro A (2001). HOXB7: a key factor for tumor-associated angiogenic switch. Cancer Res.

[21] Tu W, Zhu X, Han Y, Wen Y, Qiu G, Zhou C (2015). Overexpression of HOXB7 is associated with a poor prognosis in patients with gastric cancer. Oncol Lett.

[22] Li H, Shen LY, Yan WP, Dong B, Kang XZ, Dai L (2015). Deregulated HOXB7 Expression Predicts Poor Prognosis of Patients with Esophageal Squamous Cell Carcinoma and Regulates Cancer Cell Proliferation In Vitro and In Vivo. PLoS ONE.

[23] Nguyen Kovochich A, Arensman M, Lay AR, Rao NP, Donahue T, Li X (2013). HOXB7 promotes invasion and predicts survival in pancreatic adenocarcinoma. Cancer.

[24] Gao RT, Zhan LP, Meng C, Zhang N, Chang SM, Yao R (2015). Homeobox B7 promotes the osteogenic differentiation potential of mesenchymal stem cells by activating RUNX2 and transcript of BSP. Int J Clin Exp Med.

[25] Lv X, Li L, Lv L, Qu X, Jin S, Li K (2015). HOXD9 promotes epithelial-mesenchymal transition and cancer metastasis by ZEB1 regulation in hepatocellular carcinoma. J Exp Clin Cancer Res.

[26] Yao C, Su L, Shan J, Zhu C, Liu L, Liu C (2016). IGF/STAT3/NANOG/Slug Signaling Axis Simultaneously Controls Epithelial-Mesenchymal Transition and Stemness Maintenance in Colorectal Cancer. Stem Cells.

[27] Samardzija C, Greening DW, Escalona R, Chen M, Bilandzic M, Luwor R (2017). Knockdown of stem cell regulator Oct4A in ovarian cancer reveals cellular reprogramming associated with key regulators of cytoskeleton-extracellular matrix remodelling. Sci Rep.

[28] Candini O, Spano C, Murgia A, Grisendi G, Veronesi E, Piccinno MS (2015). Mesenchymal progenitors aging highlights a miR-196 switch targeting HOXB7 as master regulator of proliferation and osteogenesis. Stem Cells.

[29] Yuan W, Zhang X, Xu Y, Li S, Hu Y, Wu S (2014). Role of HOXB7 in regulation of progression and metastasis of human lung adenocarcinoma. Mol Carcinog.

[30] Liao WT, Jiang D, Yuan J, Cui YM, Shi XW, Chen CM (2011). HOXB7 as a prognostic factor and mediator of colorectal cancer progression. Clin Cancer Res.

[31] Zhang HF, Wu C, Alshareef A, Gupta N, Zhao Q, Xu XE (2016). The PI3K/AKT/c-MYC Axis Promotes the Acquisition of Cancer Stem-Like Features in Esophageal Squamous Cell Carcinoma. Stem Cells.

[32] Cai JQ, Xu XW, Mou YP, Chen K, Pan Y, Wu D (2016). Up-regulation of HOXB7 promotes the tumorigenesis and progression of gastric cancer and correlates with clinical characteristics. Tumour Biol.

[33] He X, Liu Z, Xia Y, Xu J, Lv G, Wang L et al. HOXB7 overexpression promotes cell proliferation and correlates with poor prognosis in gastric cancer patients by inducing expression of both AKT and MARKs. Oncotarget. 2016. doi:10.18632/oncotarget.13604. Epub ahead of print. 10.18632/oncotarget.13604 PMC5352052 27901487

[34] Ogunwobi OO, Liu C (2011). Hepatocyte growth factor up-regulation promotes carcinogenesis and epithelial-mesenchymal transition in hepatocellular carcinoma via Akt and COX-2 pathways. Clin Exp Metastasis.

[35] Liu S, Jin K, Hui Y, Fu J, Jie C, Feng S (2015). HOXB7 promotes malignant progression by activating the TGFbeta signaling pathway. Cancer Res.

[36] How C, Hui AB, Alajez NM, Shi W, Boutros PC, Clarke BA (2013). MicroRNA-196b regulates the homeobox B7-vascular endothelial growth factor axis in cervical cancer. PLoS ONE.

[37] Mayer IA, Arteaga CL (2016). The PI3K/AKT Pathway as a Target for Cancer Treatment. Annu Rev Med.

